# Kinship verification via correlation calculation-based multi-task learning

**DOI:** 10.1371/journal.pone.0329574

**Published:** 2025-09-09

**Authors:** Xiaoqian Qin, Dakun Liu, Bin Gui

**Affiliations:** 1 School of Geography and Planning, Huaiyin Normal University, Huai’an, Jiangsu, China; 2 School of Mechanical Engineering UGS College, Yancheng Institute of Technology, Yancheng, Jiangsu, China; 3 School of Computer Science and Technology, Huaiyin Normal University, Huai’an, Jiangsu, China; Government College University Faisalabad, PAKISTAN

## Abstract

Previous studies have demonstrated that metric learning approaches yield remarkable performance in the field of kinship verification. Nevertheless, a prevalent limitation of most existing methods lies in their over-reliance on learning exclusively from specified types of given kin data, which frequently results in information isolation. Although generative-based metric learning methods present potential solutions to this problem, they are hindered by substantial computational costs. To address these challenges, this paper proposes a novel correlation calculation-based multi-task learning (CCMTL) method specifically designed for kinship verification. It has been observed that kin members often exhibit a high degree of similarity in key facial organs, such as eyes, mouths, and noses. Given this similarity, similar facial features between kin members with different kin relationships frequently demonstrate certain correlations. Inspired by this observation, our proposed method aims to learn a set of metrics by leveraging both the specified kinship data and the correlations among various kinship types. These correlations are determined through an in-depth investigation of the spatial distribution relationship between the specified kinship data and other kinship types. Furthermore, we develop an efficient algorithm within the multi-task learning framework that integrates correlation exploitation with metric learning. This innovative approach effectively resolves the issue of information isolation while minimizing computational overhead. Extensive experimental validation conducted on the KinFaceW dataset demonstrates that the proposed CCMTL method achieves superior or comparable results to those of existing methods.

## Introduction

Researchers at Cornell University in the United States were the first to propose kinship verification via facial images [1], which aims to determine whether a given pair of facial images are kin-related. Here, kin-related refers to the blood relationship between parents and children. Kinship verification can be applied in social media analysis, social relationship classification, enhancing the performance of face recognition, and finding missing children [2].

Since facial image-based kinship verification shares a closely related objective with traditional face verification, researchers, in early research efforts, frequently adapted methodological frameworks from face verification to address kinship verification challenges, primarily due to the lack of specialized algorithms for familial facial analysis. This cross-disciplinary adaptation gave rise to two dominant paradigms: 1) feature extraction-based approaches [1,3], which aim to characterize kin-related facial images through handcrafted descriptors, and 2) similarity learning methods [4,5], which seek to establish a latent transformation space to capture pairwise relationships between relatives. While these pioneering methods have achieved notable success on benchmark datasets such as KinFaceW [4], their performance remains constrained by the intrinsic nonlinear manifold structure of facial data.

Fortunately, deep learning can effectively extract discriminative information relevant to labeled data from complex nonlinear datasets, as evidenced in diverse fields including face recognition [6], human-object interaction detection [7], and biomedical analysis [8–10]. Researchers have also introduced deep learning into the field of kinship verification. For instance, [11] fused different deep features. [12] proposed one deep scattering wavelet convolutional neural network. However, due to privacy concerns, the issue of small sample size in kinship verification is particularly severe. And relying solely on limited kin data of specific relationship types (e.g., father-son, father-daughter, mother-son, or mother-daughter in the KinFaceW dataset) for learning limits deep learning-based methods in two ways: 1) insufficient data volume for specified relationships causes inadequate gradient information, hindering efficient parameter updates; 2) inability to use extra discriminative information beyond the specified kin relationships, resulting in information isolation.

Recently, several studies [13–15] have adopted a generative approach to produce kin data of specific types, aiming to augment the sources of discriminative information. While these methods offer potential benefits, they incur computational overhead and pose a risk of concept drift to the algorithm, as they lack a thorough discussion on the relationship between the generated kin data and the original data. Moreover, these methods overlook information beyond specified kin data, failing to break information isolation. Therefore, developing a computationally efficient algorithm that simultaneously addresses data scarcity and information isolation constitutes a pivotal research direction in this field.

**Fig 1:**
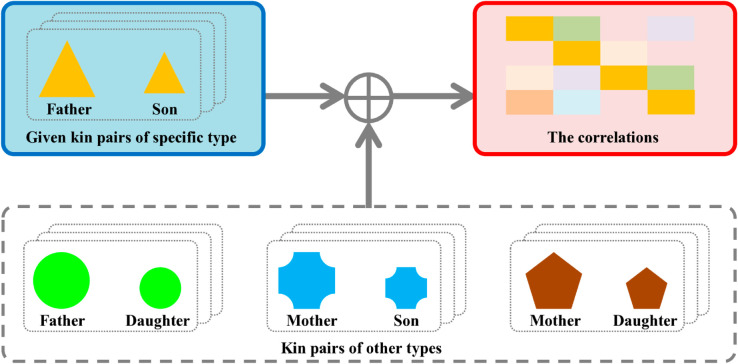
Discriminative information sources (indicated by blue and red rounded rectangles) of the proposed method.

To address this, we propose a novel correlation calculation-based multi-task learning (CCMTL) method for kinship verification. The core concept of our CCMTL method lies in effectively leveraging both the given kin pairs of specific type and the correlations among various kinship types to resolve the information isolation issue in kinship verification, as depicted in Fig 1. Our approach is motivated by prior psychological research [16], which has revealed that facial feature sets derived from all kin relationships are usually correlated, attributed to the high heritability of several key facial traits. Complementary empirical evidence in [17] further confirms the existence of such correlations among different kinship types. By emphasizing these correlations during the learning process and transforming them into auxiliary discriminative information, our method effectively resolves information isolation in kinship verification.

To this end, our contributions to kinship verification are fourfold.

First, we utilize the correlations among various kinship types as auxiliary discriminative information to boost kinship verification performance. Contrary to approaches that rely solely on kin data, incorporating such inter-kinship-type correlations in kinship verification can fundamentally address the information isolation issue. To the best of our knowledge, this is the first work that integrates the inter-kinship-type correlations within the kinship verification pipeline.

Second, instead of learning distance metrics from given and generated kin data of a specified type, we propose to learn a set of metrics by leveraging not only the specified kin data but also the inherent correlations among various kinship types. In contrast, our approach eliminates the necessity for extensive parameter tuning within deep networks, thus resulting in significantly lower computational costs.

Third, we implemented two baseline approaches to evaluate the effectiveness of our proposed correlation exploitation strategy for kinship verification. The results demonstrate that models trained on data enriched with both refined kin data and the inter-kinship-type correlations outperform those trained solely on raw kin data.

Finally, to effectively integrate correlation exploitation and metric learning, we develop an algorithm within the framework of multi-task learning. Experimental results show that our method performs favorably in kinship verification.

The remainder of this paper is organized as follows. We briefly review some of the related work in the Related work section and detail our proposed method in the Proposed methods section. Experimental results are given in the Experimental results and analysis section. We conclude this paper in the Conclusions section.

## Related work

This section briefly reviews the work related to facial image-based kinship verification, which was first proposed by Fang et al. [1]. Since then, a series of effective methods have been applied to solve this task, which can be roughly classified into feature representation-based and model-based learning methods.

Feature representation-based methods attempt to extract handcrafted or learning-based features that preserve kin-related properties from facial images. Fang et al. [1] firstly proposed utilizing local/holistic texture features to characterize facial images for facial image-based kinship verification task. Later, various features were developed to describe kin-related characteristics. [3] proposed to tackle kinship verification based on mid-level features, i.e., binary and relative attributes. [18] and [19] utilized texture color features from different color spaces and static wavelet transform features, respectively, to characterize facial images of kin relationships. Although these features have shown strong capability in capturing variations caused by uncontrollable factors such as illumination and rotation, they are still limited by their inherent nonlinear expressive power, which results in their inability to effectively characterize the similarity between parents and children. Thanks to the nonlinear representation capability of deep learning, researchers have leveraged deep neural networks( DNNs) to seek deep discriminative features. For instance, [11] fused different deep features. [12] proposed one deep scattering wavelet convolutional neural network. [20] and [21] combined shallow and handcrafted features with deep features to exploit their complementary strengths in characterizing facial images. [22] and [23] introduced the attention mechanism into deep networks. [24] utilized specific attention mechanisms to refine high-frequency and low-frequency features for more accurate local details and overall structure. In contrast, [25] utilized deep relational networks to mine multi-scale discriminative information from local regions. Furthermore, [26] proposed fusing deep face and general features through their tensor design. [27] compared the results of using different shallow features and deep features. While deep features demonstrate enhanced representational power compared to shallow counterparts, their inability to model age-progression effects in parent-child facial similarity significantly limits their capacity to capture such fine-grained similarity patterns. To tackle large divergence between the images of parent and child caused by significant age gaps, [28] presented a feature learning method, which consists of a two-stream generation-specific feature learning module and a generation-shared discrepancy reducing module. [29] and [30] respectively proposed an age-invariant adversarial feature learning module and Curvelet Transform features to address the large age gap between kin’s faces. Recently, [31] utilized deep networks to generate new facial images with different ages, ethnicities, and imaging environments to address the large divergence between the images of parent and child.

Model-based learning methods establish verification strategies to seek a feature transformation space through transfer learning [32–35] or metric learning [4]. And metric learning-based ones are frequently used in the facial image-based kinship verification task. Its primary objective is to learn a similarity metric such that the similarity between samples with kin relationship is greater than that between samples without kin relationship. Existing metric learning-based kinship verification methods can be further divided into single metric learning and multi-metric learning. NRML [4] encompasses the most representative single metric learning method and seeks only one transformation matrix, which pulls kin samples to be as close as possible and pushes non-kin samples in a neighborhood to be as far as possible. [5,36–40] learn multiple transformation matrices. Unlike the above multi-metric learning methods, [2] proposed to learn multiple local distance metrics, and one distance metric for one facial feature point. To improve nonlinear distance modeling in metric learning, researchers have integrated DNNs into metric learning frameworks. For instance, [41] and [42] proposed two deep metric learning methods that leverage hierarchical feature transformations for improved similarity measurement. And [43] developed a model called the adaptively weighted k-tuple metric network (AWk-TMN) for kinship verification, where a cross-pair metric learning loss based on k-tuple loss was introduced to capture features from multiple negative pairs. Beyond DNNs, kernel methods provide a robust alternative for nonlinear feature modeling, motivating researchers to evaluate their efficacy in kinship verification. Notably, [44] and [45] have validated the effectiveness of kernel-based metric learning methods in achieving nonlinear feature transformations that enhance discriminative power for kinship verification.

Unlike the above methods that focus solely on feature learning or metric learning, an end-to-end deep learning framework has been introduced in [46], which has jointly optimized feature learning, metric learning and meta-learning. Relatively speaking, this method, which draws inspiration from the ensemble learning concept, is more prone to achieving improved validation performance by integrating diverse models. In summary, numerous studies have been proposed for kinship verification, and a comprehensive summary of these studies can be found in a recent review article( e.g., [47]). Upon re-examining the aforementioned existing methods, we broadly divided them into two categories: those that use *existing data* and those that leverage *additional data*.

For the methods that use *existing data*, they follow the processing steps of face verification and seek discriminative features or similarity metrics from given specific type of kin data, inevitably falling into the information isolation issue. In general, these methods can make decisions, but the amount of discriminative information they can leverage is quite limited due to the scarcity of given specific type of kin data.

Relatively speaking, the methods that leverage *additional data* are more likely to utilize more discriminative information because they employed additional data. An earlier attempt was proposed in [13], wherein a generative adversarial network was utilized to generate adversarial kinship face pairs. The projection space was learned using both original and generated kinship face pairs. Recently, [14] proposed to extract feature descriptors from the generated fused image of each identity, where the fused image was a weighted linear combination of features obtained from multiple samples of the corresponding identity. And [15] presented a face age transformation model to generate facial images of various age groups to tackle age differences in the kinship images. Compared with methods that solely utilize *existing data*, these methods have the potential to use more discriminative information, thereby often achieving superior performance. However, such methods employ a generative approach to produce additional data, which not only increases the computational burden but also exposes the algorithm to the risk of concept drift due to the lack of discussion on the relationship between the generated additional data and the original data.

In this paper, we aim to tackle kinship verification task by leveraging *additional data* through a non-generative approach. Existing research has demonstrated the existence of correlations among various kinship types [17]. Furthermore, earlier psychological studies have indicated that feature sets extracted from all types of kin relationships are usually correlated, due to the high heritability of several key facial features [16]. Motivated by these findings, we propose a novel correlation calculation-based multi-task learning (CCMTL) method, which effectively exploits the correlations among various kinship types and converts them into auxiliary discriminative information, thereby mitigating the information isolation issue in kinship verification. A review of existing representative kinship verification methods is summarized in Table 1. Here, the column “Deep networks?" indicates whether the corresponding method incorporates deep neural networks, and the column “Additional data?" refers to whether the corresponding method explores discriminative information from data except given specific type of kin data (“*" denotes that the additional data originate from a non-generative approach).

**Table 1:** Review of existing representative kinship verification methods.

Focus	Year	Method	Deep networks?	Additional data?
Feature	2010	Local/holistic texture features [1]	NO	NO
	2012	Binary and relative attributes [3]	NO	NO
	2019	VGG-Face + VGG-F + LBP + HOG [11]	YES	NO
	2019	SILD +WCCN/LR [20]	YES	NO
	2019	Part-aware attention networks [22]	YES	NO
	2020	TXQDA_*WCCN*_ [21]	YES	NO
	2020	Deep relational network [25]	YES	NO
	2022	TXQEDA +WCCN [26]	YES	NO
	2022	Deep discriminant generation-shared feature learning $\left({\text{D}}^{2}\text{GFL}\right)$ [28]	YES	NO
	2022	Age-invariant adversarial feature learning (AIAF) [29]	YES	NO
	2024	Texture color features from different color spaces [18]	NO	NO
	2024	Stationary wavelet transform features [19]	NO	NO
	2024	Deep scattering wavelet convolutional neural network [12]	YES	NO
	2024	Lightweight backbone framework based on convolutional neural networks [23]	YES	NO
	2024	Curvelet Transform (CLT) features [30]	YES	NO
	2025	Frequency Feature Decoupling and Fusion Network(FDFN) [24]	YES	NO
	2022	Weighted multi sample fusion (WMSF) [14]	NO	YES
	2025	AutoSyn image synthesis framework [31]	YES	YES
Metric learning	2014	Discriminative multi-metric learning (DMML) [5]	NO	NO
	2014	Large margin multi-metric learning (${\text{LM}}^{3}\text{L}$) [36]	NO	NO
	2014	Neighborhood repulsed metric learning (NRML) [4]	NO	NO
	2015	Online similarity measure learning [48]	NO	NO
	2016	Neighborhood repulsed correlation metric learning (NRCML) [49]	NO	NO
	2016	On line similarity learning with average strategy (OSL-A) [50]	NO	NO
	2017	Sharable and individual multi-view metric learning (MvML) [38]	NO	NO
	2017	Structured sparse similarity learning $\left({\text{S}}^{3}\text{L}\right)$ [39]	NO	NO
	2017	Multi-view hybrid distance learning (MHDL3–L) [37]	NO	NO
	2017	Discriminative deep metric learning (DDMML) [41]	YES	NO
	2017	Sparse similarity metric learning (SSML) [51]	NO	NO
	2017	Local multi-metric learning (LML) [40]	NO	NO
	2018	Kinship metric learning (KML) [42]	YES	NO
	2018	Multiple kernel similarity metric (MKSM) [44]	NO	NO
	2019	Multi-view geometric mean metric learning (MvGMML) [52]	NO	NO
	2019	Weighted graph embedding based metric learning (WGEML) [45]	NO	NO
	2021	Component-based metric learning (CML) [2]	NO	NO
	2022	Adaptively weighted k-tuple metric network (AWk-TMN) [43]	NO	NO
	2019	Adversarial similarity metric learning (ASML) [13]	YES	YES
	2025	**Correlation calculation-based multi-task learning(CCMTL)**	NO	YES ( )
Hybrid	2024	Online Re-weighting Relation Network(${\text{OR}}^{2}\text{Net}$) [46]	YES	NO
	2023	Kinship verification scheme using GAN-based face age transformation [15]	YES	YES

## Proposed methods

In this section, we present our proposed kinship verification method which can exploit the correlations among various kinship types, i.e., father-son (FS), father-daughter (FD), mother-son (MS) and mother-daughter (MD). Let ${𝒟}_{q}=\{(p_{qi},c_{qi},y_{qi}){\}}_{i=1}^{M_{q}}$ be the training set of specific kinship type q(1≤q≤4), where *p*_*qi*_ and *c*_*qi*_ are two *d*-dimensional feature vectors, of the *i*-th parent and child image pair, respectively, $y_{qi}\in \{+1,-1\}$ indicates whether the *i*-th pair is parent-child relationship. We aim to learn a function f:(p,c)→{+1,−1} to check whether two previously never seen images (*p*,*c*) have a valid kin relationship.

### Kinship verification under isolated information environment

For kinship verification under isolated information environment, a separate classifier would be trained on given specific type of kin data ${𝒟}_{q}$. Let *W*_*q*_ denote the aimed transformation matrix, most existing metric learning methods take the following cost function form:

$$\min_{W_{q}}{ℒ}_{W_{q}}(\cdot )+\lambda ℛ(W_{q})$$ (1)

where ${ℒ}_{W_{q}}(\cdot )$ is the empirical loss function, $ℛ(W_{q})$ is a regulariser and *λ* is a trade-off parameter.

For ${ℒ}_{W_{q}}(\cdot )$, one can deploy a loss function that can be used to handle the binary classification problem. Hinge loss function is an important one used in kinship verification algorithm. Thus, we define ${ℒ}_{W_{q}}(\cdot )\equiv max\{0,1\;-\;yS_{W_{q}}(p,c)\}$, where $S_{W_{q}}(p,c)$ is a similarity measure function and adopts the following form [53]:

$$S_{W_{q}}(p,c)=p^{T}W_{q}c$$ (2)

To control the complexity of Eq (1) , we apply the Frobenius norm of matrix *W*_*q*_, i.e., $ℛ(W_{q})=\frac{1}{2}{\left\|W_{q}\right\|}_{F}^{2}$. The above algorithm for handling kinship verification under isolated information environment is hereinafter labeled as ‘Isolated_Learning’.

### From isolated environment to correlated information utilization

Note that the ‘Isolated_Learning’ model introduced in Eq (1) is a similarity model for specific kinship type without utilizing *additional data* except given type of kin data. In this work, we are interested in the empirical evidence presented in [17], which confirms the existence of correlations among different kinship types. These correlations can be interpreted as *additional data*, while to better model the similarity between a mother and a daughter for example, one should consider it within the context of a broader range of kinship data, i.e., instead of leading the isolated view [e.g., seeking similarity metric on facial image pairs with mother-daughter relationship] to the model design, leading the connected view [e.g., seeking similarity metric not only on facial image pairs with mother-daughter relationship but also on facial image pairs with other correlated kin relationships]. Compared with methods that introduce *additional data* through generative approaches, the proposed method offers two key advantages. On the one hand, it can fundamentally alleviate the information isolation issue, by establishing connections between kin data of the specific type and other kin data with various kinship types. On the other hand, it incurs lower computational costs and avoids the risk of concept drift, by introducing *additional data* concerning the given type of kin data into the similarity learning process in a non-generative manner.

Let us denote all kinship types as 𝒬={FS,FD,MS,MD}, given specific kinship type as q(q∈𝒬) and the correlated kinship types concerning *q* as U={u|u∈𝒬,u≠q}, respectively. In this work, each of *U* is also formulated as a bilinear function $S_{W_{u}}(p,c)$, i.e., $S_{W_{u}}(p,c)=p^{T}W_{u}c$ as in Eq (2), where *W*_*u*_ denotes the dependence structure between parent and child with kin relationship *u*. Under the viewpoint of correlated information utilization, one should learn the dependence structure *W*_*q*_ in Eq (2) for kin relationship *q* through exploiting the correlation between *W*_*q*_ and $\left\{W_{u},u\in U\right\}$. This suggests a multi-task learning procedure: learning these transformation matrices simultaneously. Consequently, we deploy a multi-task learning framework to jointly learn these transformation matrices and the relationship structures among them, which allows us to leverage the correlations between the given specific kinship type and other correlated kinship types in a non-generative manner. Specifically, we first exploit the correlations between the specific kinship type and other kinship types by analyzing their spatial positional relationships in the feature space. Subsequently, we learn a similarity measure by treating the specific kinship type and its correlated counterparts as distinct tasks within a multi-task learning framework. We detail the procedure in the following subsections.

#### Exploiting the correlations.

The primary problem is how to seek the correlated kinship types *U* concerning specific kinship type *q*. We observe that the Support Vector Data Description (SVDD) model is a one-class SVM whose boundary is solely determined by the support vector data points, capable of capturing the spatial structure of target class data, including its average characteristics and degree of dispersion. In light of this, we propose to transform the objective of seeking *U* into an investigation of the relationship between the distribution of support vectors and the centroid locations for a specific type of kinship data, in comparison to other types of kinship data.

Particularly, our algorithm has three steps.

*Step 1*: Conducting SVDD.

For kin data with relationship type *q*, we first extract the positive pairs of facial images. Then, we represent those pairs using the absolute values of the differences between the feature vectors of the parent and child. Finally, we deploy SVDD and obtain the centroid *c*_*q*_.

*Step 2*: Acquiring correlations.

For kin data with relationship type *q*, we calculate the correlation between it and kin data with other types of relationships using $r_{qu}={\left\|c_{q}\;-\;c_{u}\right\|}_{2}\;(u\in 𝒬,u\neq q)$. Thus we obtain a correlation vector $r\in R^{1\;\times \;|𝒬|-1}$.

*Step 3*: Obtaining *U*.

After conducting SVDD and calculating correlations, *r* encodes the correlations between specific kinship type *q* and other types of kin relationships. We can obtain the correlated types of kin relationships *U* concerning *q* via *r*.

Specifically, we first sort *r* in ascending order. Then, we collect the top *K* values as $\{r_{qu}{\}}_{u=1}^{K}$ and, at the same time, record the types of kin relationships corresponding to these values as *U*.

#### Learning a similarity measure.

Now, we can utilize the correlation between given type of kin relationship *q* and each of *U*. From the perspective of multi-task learning, *q* and each of *U* are treated as separate tasks but are learned jointly based on some formal definition of how they are related. In this work, motivated by the studies of human genetics [16,54], we model *W*_*q*_ and $\left\{W_{u},u\in U\right\}$ in two parts:

$$W_{q}=W_{0}+W_{q}^{\;*},\;q\in 𝒬\;\;W_{u}=W_{0}+W_{u}^{\;*},\;u\in 𝒬,u\neq q\;$$ (3)

where $W_{q}^{\;*}$ and $W_{u}^{\;*}$ are unique to the corresponding kin relationship, while all kin relationships share a common dependence structure *W*_0_. Now, we plug the above into Eq (2) and obtain the following similarity measure functions:

$$S_{W_{0},W_{q}^{*}}(p,c)=p^{T}(W_{0}+W_{q}^{\;*})c,\;q\in 𝒬\;\;S_{W_{0},W_{u}^{*}}(p,c)=p^{T}(W_{0}+W_{u}^{\;*})c,\;u\in 𝒬,u\neq q\;$$ (4)

These dependence structures are learned using the following regularized optimization problem:

$$\min_{W_{0},W_{q}^{\;*},W_{u}^{\;*}}𝒢(W_{0},W_{q}^{\;*},W_{u}^{\;*},\lambda ,\beta_{u},\gamma ,\eta )=\;\;\sum_{i=1}^{M_{q}}max\{0,1-y_{qi}S_{W_{0},W_{q}^{\;*}}(p_{qi},c_{qi})\}+\frac{\lambda }{2}{\left\|W_{q}^{\;*}\right\|}_{F}^{2}+\;\;\sum_{u=1}^{K}(\beta_{u}\sum_{i=1}^{M_{u}}max\{0,1-y_{ui}S_{W_{0},W_{u}^{\;*}}(p_{ui},c_{ui})\}+\frac{\gamma }{2}{\left\|W_{u}^{\;*}\right\|}_{F}^{2})+\frac{\eta }{2}{\left\|W_{0}\right\|}_{F}^{2}$$ (5)

where weight $\beta_{u}$ represents the degree of influence of kin relationship *u*, which is related to given type of kin relationship *q*, on the model.

Here, we believe that kin data that are spatially closer to given type of kin data are more relevant and could have a greater impact on the model. Therefore, we use the reciprocal of the previously calculated correlation vector *r* to represent the weights in Eq (5).

Based on this, we assign an extremely small value to given type of kin data itself, take its reciprocal as the weight for the first term of Eq (5), and then normalize it with $\{\beta_{u}{\}}_{u=1}^{K}$. Now, we can combine and rewrite the first three terms of Eq (5) to obtain the final optimization objective:

$$\min_{W_{0},W_{v}^{\;*},\beta_{v}}ℱ=\sum_{v=1}^{K+1}(\beta_{v}\sum_{i=1}^{M_{v}}max\{0,1-y_{vi}S_{W_{0},W_{v}^{\;*}}(p_{vi},c_{vi})\}+\frac{\lambda }{2}{\left\|W_{v}^{\;*}\right\|}_{F}^{2})+\frac{\eta }{2}{\left\|W_{0}\right\|}_{F}^{2}\;subject\;to\;\sum_{v=1}^{K+1}\beta_{v}=1,0<\beta_{v}<1\;$$ (6)

#### Performing metric learning.

To estimate the parameters in Eq (6), i.e., the transformation matrices *W*_0_, $\{W_{v}^{\;*}{\}}_{v=1}^{K+1}$ and the weights $\{\beta_{v}{\}}_{v=1}^{K+1}$, we adopt an alternating iteration strategy to solve it.

First, we update *W*_0_ by fixing other parameters. In this situation, Eq (6) is equivalent to the following expression:

$$\min_{W_{0}}ℱ=\sum_{v=1}^{K+1}(\beta_{v}\sum_{i=1}^{M_{v}}max\{0,1-y_{vi}S_{W_{0},W_{v}^{\;*}}(p_{vi},c_{vi})\}+\frac{\lambda }{2}{\left\|W_{v}^{\;*}\right\|}_{F}^{2})+\frac{\eta }{2}{\left\|W_{0}\right\|}_{F}^{2}$$ (7)

To obtain *W*_0_, we employ the stochastic sub-gradient descent method to solve Eq (7). And the gradient of objective function ℱ with respect to *W*_0_ can be calculated as:

$$\frac{\partial F}{\partial W_{0}}=\left\{ \begin{array}{c} \eta W_{0},\;S_{W_{0},W_{v}^{\;*}}(p_{vi},c_{vi})\geq 1\\ -\beta_{v}y_{vi}p_{vi}c_{vi}+\eta W_{0},\;S_{W_{0},W_{v}^{\;*}}(p_{vi},c_{vi})<1 \end{array}\right.$$ (8)

Now, one can update *W*_0_ by using

$$W_{0}=W_{0}-\alpha \frac{\partial F}{\partial W_{0}}\;$$ (9)

where *α* is the learning rate.

Then, we update $W_{v}^{\;*}$ by fixing others. Similar to *W*_0_, we can update $W_{v}^{\;*}$ by using:

$$W_{v}^{\;*}=W_{v}^{\;*}-\alpha \frac{\partial F}{\partial W_{v}^{\;*}}$$ (10)

where $\frac{\partial F}{\partial W_{v}^{\;*}}$ can be computed as:

$$\frac{\partial F}{\partial W_{v}^{\;*}}=\left\{ \begin{array}{c} \lambda W_{v}^{*},\;S_{W_{0},W_{v}^{\;*}}(p_{vi},c_{vi})\geq 1\\ -\beta_{v}y_{vi}p_{vi}c_{vi}+\lambda W_{v}^{*},\;S_{W_{0},W_{v}^{\;*}}(p_{vi},c_{vi})<1 \end{array}\right.$$ (11)

Finally, we update $\beta_{v}$ with other new parameters. For this, we construct a Lagrange function as follows:

$$L=\sum_{v=1}^{K+1}(\beta_{v}\sum_{i=1}^{M_{v}}max\{0,1-y_{vi}S_{W_{0},W_{v}^{\;*}}(p_{vi},c_{vi})\}+\frac{\lambda }{2}{\left\|W_{v}^{\;*}\right\|}_{F}^{2})+\frac{\eta }{2}{\left\|W_{0}\right\|}_{F}^{2}-\theta (\sum_{v=1}^{K+1}\beta_{v}-1)\;\;$$ (12)

where *θ* is a Lagrange multiplier. We calculate the derivative of *L* in Eq (12) with respect to $\beta_{v}$ and obtain:

$$\frac{\partial L}{\partial \beta_{v}}=\sum_{i=1}^{M_{v}}max\{0,1-y_{vi}S_{W_{0},W_{v}^{\;*}}(p_{vi},c_{vi})\}-\theta$$ (13)

Now, we can update $\beta_{v}$ by using

$$\beta_{v}=\beta_{v}-\alpha \frac{\partial L}{\partial \beta_{v}}$$ (14)

We repeat the above steps until the algorithm meets a specific convergence criterion. The detailed process of our proposed CCMTL algorithm is summarized in Algorithm 1.

**Algorithm 1. CCMTL.**

**Input:**

Training set: ${𝒟}_{q}=\{(p_{qi},c_{qi},y_{qi}){\}}_{i=1}^{M_{q}}$ be the given type of

kin relationship q(q∈𝒬);

${𝒟}_{u}=\{(p_{ui},c_{ui},y_{ui}){\}}_{i=1}^{M_{u}}$ be the other types of

kin relationships (u∈𝒬,u≠q);

Parameters: trade-off parameters *λ* and *η*, the number of

correlated kin types *K*, learning rate *α*, Lagrange multiplier

*θ*, iteration number *Nu*, and convergence error *τ*

**Output:**

The correlated types of kin relationships *U* concerning given

type *q*;

The transformation matrices *W*_0_ and $\{W_{v}^{*}{\}}_{v=1}^{K+1}$;

The correlation weights $\{\beta_{v}{\}}_{v=1}^{K+1}$;

1: **Step1: Exploiting correlated information:**

2: **Step1.1: Conducting SVDD:**

3:    For kin relationship *q* and other types of kin

relationships {u|u∈𝒬,u≠q}, repeat:

4:       Extract the positive pairs of facial images;

5:       Represent pairs using abs. values of diff.

between parent and child feature vectors;

6:       Deploy SVDD and obtain centroids, i.e., *c*_*q*_ and

$\left\{c_{u}|u\in 𝒬,u\neq q\right\}$;

7: **Step1.2: Acquiring correlations:**

8:    For each other type of kinship {u|u∈𝒬,u≠q}, repeat:

9:       Compute the correlation using $r_{qu}={\left\|c_{q}-c_{u}\right\|}_{2}$;

10:    Obtain a correlation vector $r\in R^{1\;\times \;|𝒬|-1}$;

11: **Step1.3: Obtaining the correlated kinship types *U*:**

12:    Sort the correlation vector *r* in ascending order;

13:    Collect the top *K* values as $\{r_{qu}{\}}_{u=1}^{K}$;

14:    Record the types of kin relationships corresponding to

these values as *U*;

15: **Step2: Learning a similarity:**

16: **Step2.1: Initilization:**

17:    Set $W_{0}=I$, $\{W_{v}^{*}{\}}_{v=1}^{K+1}=I$;

18:    Randomly Generate an extremely small number *s* ;

19:    Normalize 1/*s* and $\{1/r_{qu}{\}}_{u=1}^{K}$ together and set them as

the weights $\{\beta_{v}{\}}_{v=1}^{K+1}$ in Eq (6);

20: **Step2.2: Local optimization:**

21:    For r=1,2,...,Nu, repeat

22:       Randomly select a pair of samples $\left(p_{qi},c_{qi},y_{qi}\right)$ from

${𝒟}_{q}$;

23:       Compute the gradient in Eq (8);

24:       Update *W*_0_ by using Eq (9);

25:       for v=1,2,...,K+1 do

26:         Compute the gradient in Eq (11);

27:         Update $W_{v}^{*}$ by using Eq (10);

28:       end

29:       for v=1,2,...,K+1 do

30:         Compute the gradient in Eq (13);

31:         Update $\beta_{v}$ by using Eq (14);

32:       end

33:       Calculate *F*_*r*_ according to Eq (6);

34:       if *r* > 2 and $|F_{r}-F_{r-1}|<\tau$, go to Step3.

35: **Step3: Output:**

36:    Output *U*, *W*_0_, $\{W_{v}^{*}{\}}_{v=1}^{K+1}$ and $\{\beta_{v}{\}}_{v=1}^{K+1}$.

Having obtained our CCMTL model, we apply it to each pair to measure the similarity using Eq (4). We then train an SVM classifier to predict whether each test pair has a kin relationship or not.

## Experimental results and analysis

In this section, experimental evaluations for the proposed method are conducted on the largest publicly available kinship face image dataset, namely, KinFaceW [4]. We detail the experimental settings and results in the following subsections.

### Dataset and experimental settings

KinFaceW consists of two subsets: KinFaceW-I and KinFaceW-II. Both subsets include four kin relationships: Father-Son (FS), Father-Daughter (FD), Mother-Son (MS), and Mother-Daughter (MD). The number of different kin pairs in these subsets can be found in S1 Table. The key difference is that KinFaceW-I pairs are from different photos, while KinFaceW-II pairs are from the same photo.

For this dataset, we employ each aligned facial image of resolution 64×64 for feature extraction. For each image, we derive two distinct types of feature representations:

1) Local Binary Patterns (LBP) [55]: We partition each image into 4×4 non-overlapping blocks, each of size 16×16. A 256-dimensional uniform LBP descriptor is computed for each block, and these descriptors are concatenated to produce a 4096-dimensional feature vector.

2) Scale-Invariant Feature Transform (SIFT) [56]: The image is divided into overlapping patches arranged in a 7×7 grid, with each patch measuring 16×16 pixels. SIFT descriptors are extracted from each patch and concatenated to form a 6272-dimensional feature vector.

Furthermore, we apply PCA to reduce the dimensionality of both feature vectors to 100 dimensions.

In our experimental setup, we adhere to the evaluation protocol outlined in [4] and implement a 5-fold cross-validation strategy to ensure fair comparisons. The final performance metric utilized for evaluation is the mean verification accuracy.

For the parameters of our method, we used 4-fold cross-validation to determine optimal values from predefined ranges. Specifically, *λ*, *η*, *α* and *θ* were tuned within $\left\{1\;\times \;10^{-4}:10:1\;\times \;10^{4}\right\}$ and *K* within {1,2,3}. We empirically set τ=0.1 and *N*_*u*_ = 200 for model scaling. Furthermore, we fine-tuned the hyper-parameters of SVDD, namely C and Gamma, to optimize performance.

### Experimental results

#### Baseline results.

To assess the efficacy of our proposed correlated information utilization strategy for kinship verification, we implemented two baseline methods. Specifically, we used Isolated_Learning as one baseline, employing Eq (1) and Eq (2). Additionally, we applied Isolated_Learning to the training data that had undergone SVDD filtering, and designated this approach as ‘Isolated_Learning+SVDD’.

Tables 2 and 3 compare the results of our method with two above baselines on the KinFaceW-I and KinFaceW-II datasets. The observations are as follows:

**Table 2:** Comparisons (%) of our proposed method with two baselines on the KinFaceW-I dataset.

Method	Feature	FS	FD	MS	MD	Mean
Isolated_Learning	LBP	65.0	66.2	60.0	66.3	64.4
	SIFT	71.0	64.6	65.1	61.2	65.5
Isolated_Learning+SVDD	LBP	67.2	71.4	60.3	75.0	68.5
	SIFT	74.9	73.2	70.4	68.3	71.7
CCMTL (*proposed*)	LBP	74.6	79.7	74.8	76.4	76.4
	SIFT	80.3	78.5	77.6	76.5	78.2

**Table 3:** Comparisons (%) of our proposed method with two baselines on the KinFaceW-II dataset.

Method	Feature	FS	FD	MS	MD	Mean
Isolated_Learning	LBP	66.0	67.3	68.0	65.0	66.6
	SIFT	68.2	68.4	69.7	67.2	68.4
Isolated_Learning+SVDD	LBP	71.2	70.6	72.0	69.3	70.8
	SIFT	76.5	71.4	71.0	70.0	72.2
CCMTL (*proposed*)	LBP	78.3	77.0	79.1	76.0	77.6
	SIFT	82.7	76.3	77.9	78.1	78.8

1) Isolated_Learning exhibits the lowest performance on both subsets. In contrast, Isolated_Learning+SVDD achieves average improvements of 4.1% and 6.2% across four kin relationships in the KinFaceW-I dataset, and improvements of 4.2% and 3.8% on the four relationships in the KinFaceW-II dataset, respectively. These improvements demonstrate the effectiveness of incorporating the SVDD model. Furthermore, the proposed CCMTL method further enhances performance, illustrating the efficacy of mining and leveraging correlations among diverse kinship types. To further illustrate this, we visualize the learned correlations on the KinFaceW dataset in S1 Fig. We can observe that the correlation between FD and MS is the highest, while that between FS and MD is the lowest, and such information is effectively captured and utilized by our model.

2) LBP and SIFT can yield promising performance. This demonstrates the effectiveness of these two types of features, which is consistent with some previous kinship verification results [4,13]. When integrated with SIFT, our proposed method, along with two baseline approaches, demonstrates superior average performance compared to when combined with LBP. This is primarily because SIFT exhibits superior robustness to scale changes, rotations, and illumination variations, making it more suitable for characterizing the variable similarities between parent-child pairs.

To better emphasize the effectiveness of our proposed correlated information utilization strategy and illustrate the differences between two feature representation methods, the receiver operating characteristic(ROC) curves of different methods are presented in S2 Fig. As evidenced by these figures, our proposed CCMTL model, which incorporates the correlations among various kinship types, significantly outperforms the two baseline approaches in terms of performance improvement. Furthermore, the SIFT feature yields the best performance, as indicated by the ROC curves.

#### Comparison with existing multi-task learning methods.

We conducted empirical comparisons with two competing methods in the realm of subspace regularized multi-task learning: No-group MTL [57], which assumes a low-dimensional subspace shared by all interrelated task parameters, and GO-MTL [58], a recently proposed method assuming each task parameter vector can be expressed as a linear combination of a finite set of basis tasks. Additionally, for both of these methods, we developed variant algorithms that utilized data filtered through SVDD, termed ‘No-group MTL [57] +SVDD’ and ‘GO-MTL [58] +SVDD’, respectively.

Table 4 summarizes the results. The observations are as follows:

**Table 4:** Mean verification accuracy (%) of different methods with SIFT on the KinFaceW dataset.

Method	KinFaceW-I	KinFaceW-II
No-group MTL [57]	69.2	72.0
No-group MTL [57]+SVDD	73.0	74.2
GO-MTL [58]	71.6	73.0
GO-MTL [58]+SVDD	74.9	76.2
Isolated_Learning+SVDD	71.7	72.2
CCMTL (*proposed*)	78.2	78.8

1) The SVDD versions of two competing multi-task learning methods outperform Isolated_Learning+SVDD, once again indicating that mining and utilizing the correlations among various kinship types can effectively improve generalization performance in kinship verification.

2) Both competing methods achieve higher performance when learning on data filtered by SVDD, demonstrating the effectiveness of the SVDD model.

3) By introducing an overlapping group structure, GO-MTL [58]+SVDD improves upon No-group MTL [57]+SVDD by 1.9% and 2.0% on KinFaceW-I and KinFaceW-II, respectively. This suggests that there is a certain structure between the kinship type to be learned and other correlated kinship types. Furthermore, the proposed CCMTL method achieves additional improvements of 3.3% and 2.6% over GO-MTL [58]+SVDD on two datasets. The reason is that, compared to the overlapping group structure assumed by GO-MTL [58]+SVDD, the proposed CCMTL method shares discriminative information across tasks via a common transformation matrix in a flexible manner, potentially leading to higher performance.

#### Comparison with existing metric learning methods.

Given that the proposed method necessitates learning transformation matrices, which aligns with the learning objectives of existing metric learning methods such as NRML [4], as well as its modified versions, NRCML [49] and PDFL+NRML [59], we further compared our method with these three metric learning approaches on the KinFaceW dataset. The performance of all comparison methods was sourced from the corresponding literature.

Tables 5 and 6 present the results on KinFaceW-I and KinFaceW-II, respectively. We can observe that PDFL+NRML [59] achieves the highest average performance among all compared methods. This superiority is attributed to its utilization of an intermediate-level discriminative feature learned from the LFW dataset, which can capture the parent-child similarity characteristics more accurately than the low-level features utilized by other methods. Compared to PDFL+NRML [59], the proposed CCMTL method, leveraging LBP and SIFT features, achieves average improvements of 9.0% and 10.8% across the four relationships in KinFaceW-I, and 0.1% and 1.3% on KinFaceW-II, respectively. These results further validate the effectiveness of our proposed method.

**Table 5:** Comparisons (%) of our proposed method with three metric learning approaches on the KinFaceW-I dataset.

Method	Feature	FS	FD	MS	MD	Mean
NRML [4]	LBP	64.7	65.2	59.4	65.4	63.7
	SIFT	70.5	64.0	64.0	60.4	63.8
NRCML [49]	LBP	66.7	67.2	61.4	67.4	65.7
	SIFT	72.5	66.0	66.0	62.4	65.8
PDFL+NRML [59]	Raw Pixels	N/A	N/A	N/A	N/A	67.4
CCMTL (*proposed*)	LBP	74.6	79.7	74.8	76.4	76.4
	SIFT	80.3	78.5	77.6	76.5	78.2

**Table 6:** Comparisons (%) of our proposed method with three metric learning approaches on the KinFaceW-II dataset.

Method	Feature	FS	FD	MS	MD	Mean
NRML [4]	LBP	69.0	69.5	69.8	69.0	69.5
	SIFT	69.0	60.9	60.8	61.4	62.8
NRCML [49]	LBP	72.0	72.5	72.8	72.0	72.5
	SIFT	71.0	63.9	63.8	64.4	65.8
PDFL+NRML [59]	Raw Pixels	N/A	N/A	N/A	N/A	77.5
CCMTL (*proposed*)	LBP	78.3	77.0	79.1	76.0	77.6
	SIFT	82.7	76.3	77.9	78.1	78.8

**Fig 2:**
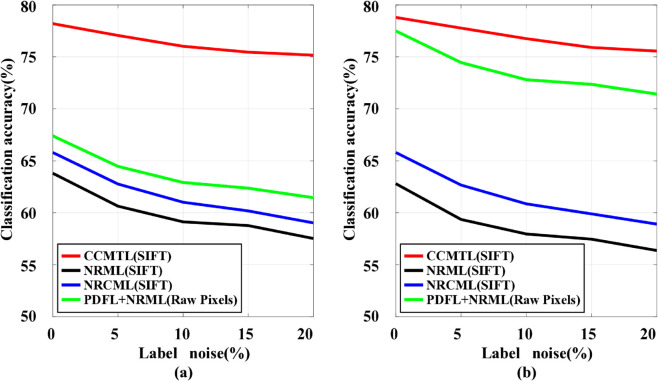
Mean verification accuracy of different methods with varying degree of label noise. (a) On the KinFaceW-I dataset. (b) On the KinFaceW-II dataset.

Furthermore, given the inevitable presence of noise in images captured in natural settings, it is crucial to assess the robustness of learning models against such noise. To this end, we artificially introduced 5%-20% label noise into the training data by randomly altering the labels of the training samples. It should be noted that the labels of the test samples remained unchanged during this process. We conducted ten repeated experiments for both our proposed method and the comparison methods, and compared their average performances. Fig 2 presents the experimental results. One can see that the performance of all methods gradually deteriorates with increasing noise levels. With the continuous addition of noise, the performance of all compared metric learning methods decreases significantly, whereas the proposed method still achieves better performance in such challenging scenarios, indicating its noise robustness.

#### Comparison with state-of-the-art methods.

We also conducted a comparative analysis of our proposed method against several state-of-the-art learning approaches: feature-based Fusion [18], SILD+WCCN/LR [20], FDFN [24] and CLT [30], deep learning-based ASML [13] and KML [42], metric learning-based TXQDA [60], and hybrid-based ${\text{OR}}^{2}\text{Net}$ [46]. More specifically, Fusion [18] utilizes texture color features from different color spaces, SILD+WCCN/LR [20] combines deep and shallow features, FDFN [24] introduces a deep learning framework that decouples features into high and low-frequency components and subsequently fuses them using attention mechanisms, CLT [30] leverages Curvelet Transform features and a CNN-based feature selection process, ASML [13] introduces generative adversarial concepts into kinship verification, KML [42] employs a pair of deep neural networks to learn a deep transformation projection space, TXQDA [60] extends cross-quadratic discriminant analysis (XQDA) by leveraging tensors to preserve kin data, and ${\text{OR}}^{2}\text{Net}$ [46] jointly optimizes feature learning, metric learning and meta-learning. The performances of all comparison methods are sourced from the corresponding publications.

Table 7 presents the mean verification accuracy of our proposed CCMTL method and four other learning methods on the KinFaceW dataset. Observations are summarized as follows:

**Table 7:** Comparisons (%) of our proposed method with state-of-the-art methods on the KinFaceW dataset.

Foucs	Method	Feature	KinFaceW-I	KinFaceW-II
Feature	CLT [30]	Curvelet Transform features+	81.0	76.9
		feature selection		
	SILD+WCCN/LR [20]	Deep+Shallow	N/A	86.2
	FDFN [24]	Deep	81.3	90.6
	Fusion [18]	Features from different color spaces	79.5	90.7
Deep learning	ASML [13]	LBP	77.1	78.6
	KML [42]	SIFT	78.7	81.0
Metric learning	TXQDA [60]	MSLPQ(3+5+7+9+11)+	N/A	87.2
		MSBSIF(3+5+7+9+11)		
Hybrid	${\text{OR}}^{2}\text{Net}$ [46]	Deep	84.7	94.6
Multi-task learning	CCMTL (*proposed*)	LBP	76.4	77.6
		SIFT	78.2	78.8

1) ASML [13] is closest to the proposed CCMTL method because both learn feature transformation matrices based on traditional manual features. We can observe that the proposed CCMTL method can achieve comparable performance to ASML, demonstrating its effectiveness. However, it is noteworthy that the projection space obtained by ASML [13] is learned from both original and GAN-generated kin data. In contrast, the proposed CCMTL method learns from existing kin data only, which not only incurs a lower computational cost but also mitigates the risk of concept drift.

2) The other comparison methods outperform CCMTL, primarily due to their utilization of multi-view, multi-scale or deep network techniques. More specifically, Fusion [18] utilizes texture color features from different color spaces and TXQDA [60] leverages two local texture descriptors at multiple scales. These two methods have achieved the two highest performance levels among all methods that utilize shallow features. This demonstrates the importance of leveraging multi-view and multi-scale discriminative information in kinship verification, as they enable the capture of fine-grained similarities between parent-child pairs that single-view or single-scale features cannot. SILD+WCCN/LR [20] and FDFN [24] leverage features extracted through deep neural networks. All two methods have achieved remarkably high performance. This emphasizes the significance of deep networks’ nonlinear modeling in kinship verification, since face images lie in nonlinear manifolds. Note that the proposed method focuses on leveraging correlations among diverse kinship types in the multi-task learning framework, thus only adopting widely-used shallow features. Additionally, it can be combined with multi-view, multi-scale and deep features, potentially improving its performance.

3) Notably, ${\text{OR}}^{2}\text{Net}$ [46] achieves the highest performance, attributable to several factors. To start, it concatenates multi-scale features extracted from different convolutional blocks and constructs a Family ID Module to learn more discriminative representations. Moreover, it incorporates a deep nonlinear metric learning process to better capture the underlying relationships within kin data. Additionally, a meta re-weighting network is constructed to ensure algorithm robustness. In contrast to other approaches that focus solely on feature learning, metric learning, or deep learning, the hybrid ${\text{OR}}^{2}\text{Net}$ [46] is more likely to achieve superior performance. This is because it formulates a comprehensive solution by integrating the strengths of these individual learning paradigms.

#### Parameter analysis.

Since the proposed CCMTL method involves the selection of the number of correlated kinship types *K*, we also investigated the algorithm’s performance across various *K* values.

**Fig 3:**
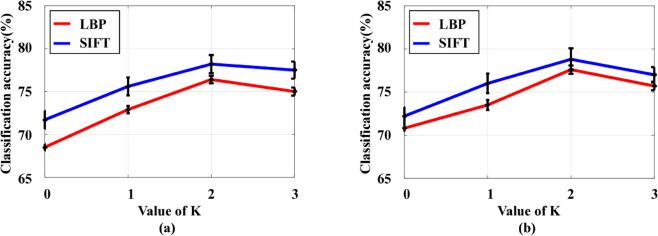
Mean verification accuracy of our proposed method versus different values of parameter K. (a) On the KinFaceW-I dataset. (b) On the KinFaceW-II dataset.

Fig 3 illustrates the average performance of our proposed method versus different values of *K* on the KinFaceW dataset. Here, *K* = 0 corresponds to the ‘Isolated_Learning’ scenario. We can observe that the proposed method exhibits the lowest performance under this setting. As K increases, the performance of the proposed method improves due to the model’s capacity to leverage correlation information from diverse related kinship types, thereby enhancing its discriminative capabilities. However, when *K* attains its maximum value, the performance of the proposed method declines slightly, which is attributed to the interference from low-related kinship types during the model’s learning process. Therefore, we recommend setting *K* = 2 in practical applications.

### Discussion

In this subsection, we compare our proposed CCMTL method with existing metric learning-based kinship verification approaches, emphasizing two key differences.

- Whether the correlations among various kinship types have been fully utilized. Existing approaches solely focus on extracting discriminative information from given or generated gender-fixed kinship facial images. In contrast, our proposed method goes beyond this by leveraging given kinship facial images and exploring the correlations among various kinship types, where the correlations are transformed into auxiliary discriminative information to enhance kinship verification performance. Tables 2, 3 and 4 show the superiority of our proposed method, highlighting the effectiveness of utilizing correlations.
- Whether the influence of noise has been considered. Facial analysis algorithms often suffer from noise in uncontrolled environments. Unlike existing kinship verification approaches that rarely focus on noise, our proposed method employs SVDD for preprocessing to enhance robustness. Fig 2 illustrates our method’s superior robustness across noise levels.

Additionally, deep learning-based methods [13,20,42] have been proposed for kinship verification, leveraging the strong performance of CNN models. Despite the potential of CNNs for kinship verification, the proposed method does not utilize CNNs due to several reasons.

Firstly, learning with CNN typically requires a large number of training samples, which are extremely expensive to collect in kinship verification due to the privacy concerns, involved time and human costs. This limitation is echoed in prior work, such as [61], which pre-trains on large face verification datasets before fine-tuning for kinship verification. Recently, several studies [13–15] have adopted generative-based approaches to produce kin pairs, aiming to augment the number of training samples. Relatively speaking, the proposed method can achieve comparable performance without the use of CNN, thus being more cost-effective. Secondly, directly applying state-of-the-art CNNs to kinship verification is not ideal due to the large divergence between the images of parent and child, which heavily relies on prior knowledge. This is supported by research [62] that incorporates scene context into deep neural networks using knowledge graphs and graph reasoning models. In contrast, the proposed method utilizes the correlations among various kinship types through simpler vector operations, and therefore being more practical. This superiority is demonstrated in Table 7.

In the future, we can explore the integration of the correlations among various kinship types into deep neural networks to fully leverage the classification capabilities of CNNs.

## Conclusions

Facial image-based kinship verification has a wide range of applications, such as social media analysis, social relationships classification, and missing children identification. However, current methods face significant limitations in effectively characterizing the facial similarity between parents and children. This paper introduces a novel correlation calculation-based multi-task learning (CCMTL) method for kinship verification. The proposed CCMTL learns a set of metrics from both the specified kinship data and the correlations among diverse related kinship types within a multi-task learning framework. On the one hand, by incorporating inter-kinship-type correlations, the proposed method can fundamentally resolve the information isolation issue that existing approaches face. On the other hand, by utilizing these correlations as additional discriminative information in a non-generative manner, the proposed method eliminates the necessity for extensive parameter tuning within deep networks, thereby reducing computational costs.

Experimental results have demonstrated the effectiveness of the proposed method, and they also advocate the incorporation of inter-kinship-type correlations into kinship verification to enable more efficient training and deployment, particularly when computational resources are limited. In future work, one can construct a unified framework that integrates correlation utilization, feature learning, and metric learning to further improve the performance of kinship verification.

## Supporting information

S1 Table The number of different kin pairs in the KinFaceW dataset. (DOCX)

S1 Fig Illustration of the learned correlations on the KinFaceW dataset. Each rectangular color block visually encodes the strength of the correlation between the kin relationships designated by the corresponding row and column. The stronger the correlation, the smaller the numerical value, and the color block tends towards blue; conversely, the weaker the correlation, the larger the numerical value, and the color block tends towards red.
(TIF)

S2 Fig The ROC curves of different methods. (a) On the KinFaceW-I dataset. (b) On the KinFaceW-II dataset.
(TIF)
