# Supplementary material for: Kinship verification via correlation calculation-based multi-task learning
Source: PLoS One. 2025 Sep 9;20(9):e0329574. doi: 10.1371/journal.pone.0329574 (PMC12419595; doi:10.1371/journal.pone.0329574)
Supplement: S1 Table — (DOCX) [file pone.0329574.s001.docx]

S1 Table The number of different kin pairs in the KinFaceW dataset.

|  | **Kin relationship type** | | | |
| --- | --- | --- | --- | --- |
|  | **FS** | **FD** | **MS** | **MD** |
| **KinFaceW-I** | 156 | 134 | 116 | 127 |
| **KinFaceW-II** | 250 | 250 | 250 | 250 |
