# Supplementary figures and images for: Kinship verification via correlation calculation-based multi-task learning
Source: PLoS One. 2025 Sep 9;20(9):e0329574. doi: 10.1371/journal.pone.0329574 (PMC12419595; doi:10.1371/journal.pone.0329574)

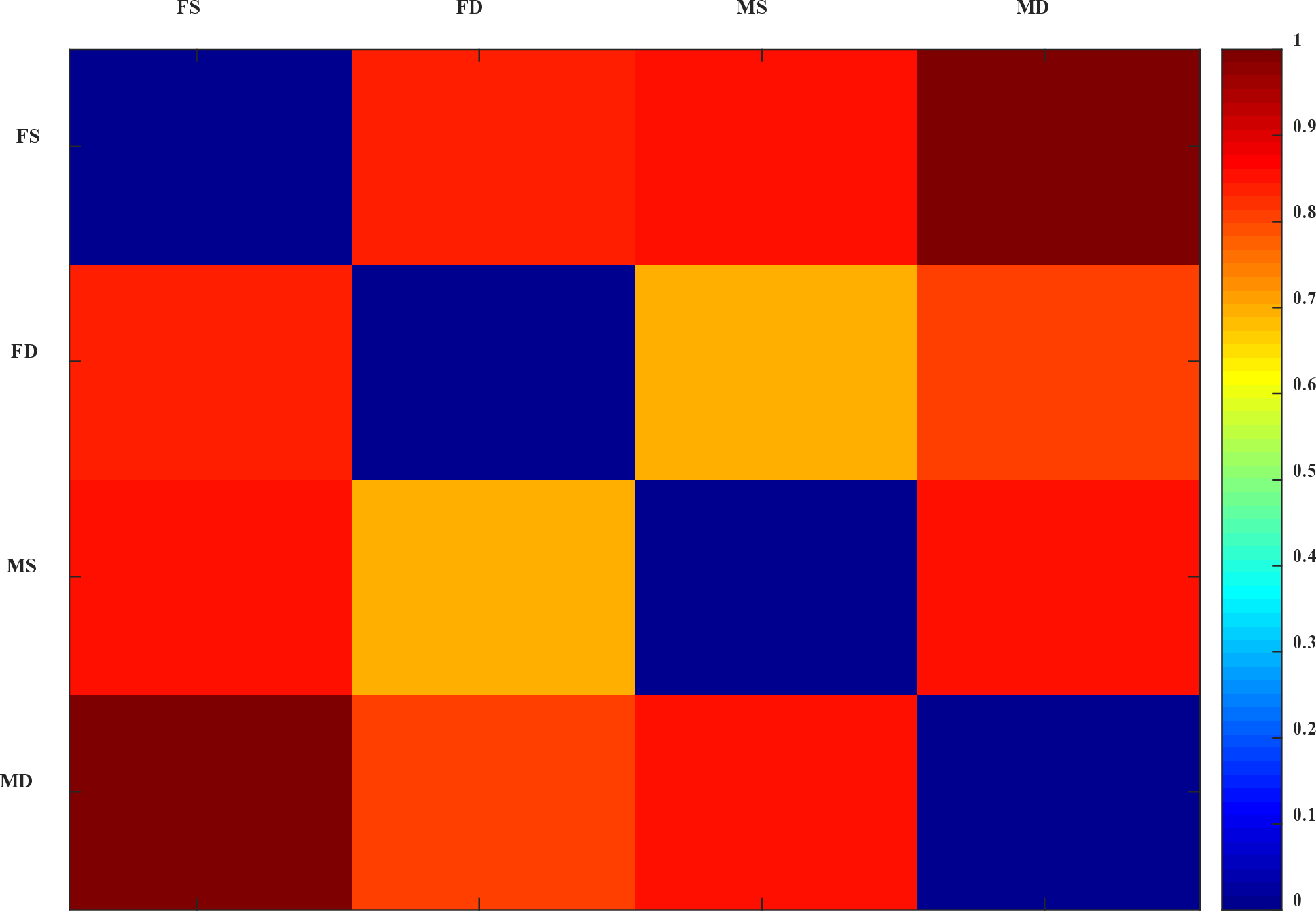

Supplement: S1 Fig — Each rectangular color block visually encodes the strength of the correlation between the kin relationships designated by the corresponding row and column. The stronger the correlation, the smaller the numerical value, and the color block tends towards blue; conversely, the weaker the correlation, the larger the numerical value, and the color block tends towards red. (TIF) [file pone.0329574.s002.tif]

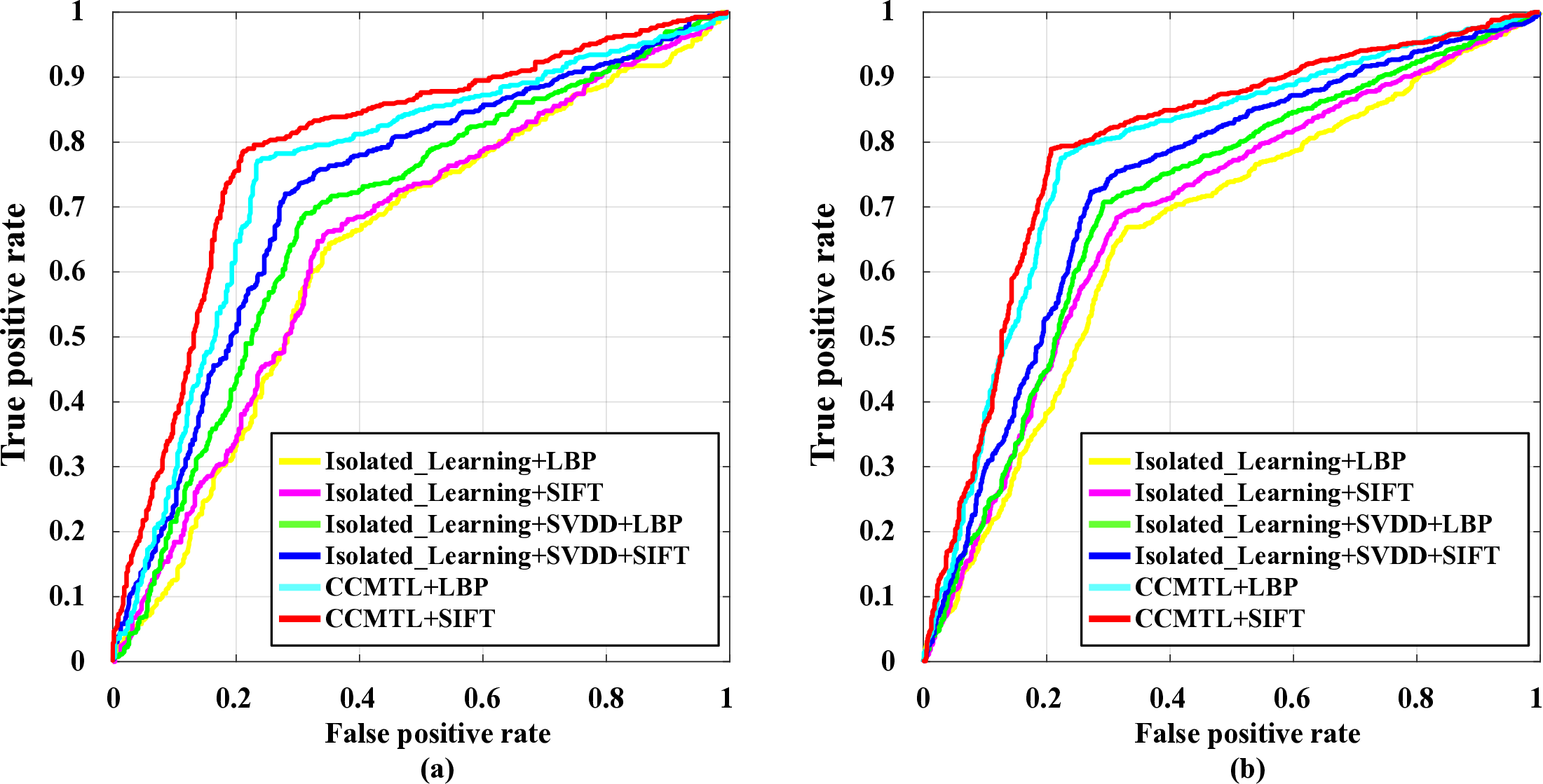

Supplement: S2 Fig — (a) On the KinFaceW-I dataset. (b) On the KinFaceW-II dataset. (TIF) [file pone.0329574.s003.tif]
